# Neural Network Pattern Recognition of Ultrasound Image Gray Scale Intensity Histograms of Breast Lesions to Differentiate Between Benign and Malignant Lesions: Analytical Study

**DOI:** 10.2196/23808

**Published:** 2021-06-02

**Authors:** Arivan Ramachandran, Shivabalan Kathavarayan Ramu

**Affiliations:** 1 Postgraduate Institute of Medical Education and Research Chandigarh India; 2 Mahatma Gandhi Medical College and Research Institute Puducherry India; 3 All India Institute of Medical Sciences New Delhi India

**Keywords:** radiology, imaging, neural network, images

## Abstract

**Background:**

Ultrasound-based radiomic features to differentiate between benign and malignant breast lesions with the help of machine learning is currently being researched. The mean echogenicity ratio has been used for the diagnosis of malignant breast lesions. However, gray scale intensity histogram values as a single radiomic feature for the detection of malignant breast lesions using machine learning algorithms have not been explored yet.

**Objective:**

This study aims to assess the utility of a simple convolutional neural network in classifying benign and malignant breast lesions using gray scale intensity values of the lesion.

**Methods:**

An open-access online data set of 200 ultrasonogram breast lesions were collected, and regions of interest were drawn over the lesions. The gray scale intensity values of the lesions were extracted. An input file containing the values and an output file consisting of the breast lesions’ diagnoses were created. The convolutional neural network was trained using the files and tested on the whole data set.

**Results:**

The trained convolutional neural network had an accuracy of 94.5% and a precision of 94%. The sensitivity and specificity were 94.9% and 94.1%, respectively.

**Conclusions:**

Simple neural networks, which are cheap and easy to use, can be applied to diagnose malignant breast lesions with gray scale intensity values obtained from ultrasonogram images in low-resource settings with minimal personnel.

## Introduction

Breast cancer is the most common cancer in Indian women with a prevalence of 25.8 per 100,000. Lack of adequate breast cancer screening, diagnosis at a later stage, and unavailability of resources are quoted as the main reasons for the increase in mortality in patients with breast cancer in India [1]. The breast cancer mortality in South Asia increased from 6.12 to 9.14 per 100,000 according to a 25-year study [2]. Multiple imaging modalities like ultrasonogram, x-ray mammography, computed tomography, positron emission tomography, and magnetic resonance imaging are being used to screen, diagnose, and evaluate breast cancer.

Ultrasound is one of the basic radiological imaging modalities available in hospitals and it is the imaging modality of choice in suspicious breast lesions in young women and pregnant women. Ultrasound has higher accuracy and sensitivity in the detection of malignant lesions compared to x-ray mammography [3]. Even with higher accuracy of ultrasonograms, the presence of significant interobserver variability is a notable disadvantage of ultrasonograms. This problem can be solved using radiomics-based diagnostic methods since it standardizes the substantial amount of data available for diagnosis [4].

Application of artificial intelligence for image recognition and classification is an upcoming method and can be implemented in areas with resource and personnel limitations, as it is suggested that neural network–based differentiation of breast lesions has the capacity to substantially reduce unnecessary biopsies and can perform equivalent to trained human radiologists [5,6]. In this study, we are evaluating the efficiency of convolutional neural networks (CNNs) in classifying malignant and benign breast ultrasonogram images downloaded from an online data set based on their gray scale intensity histograms.

## Methods

**Figure 1:**
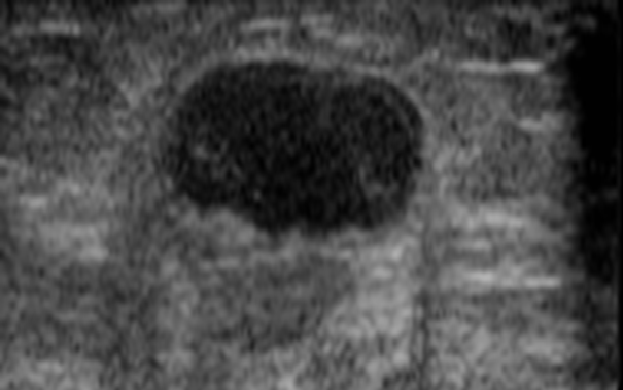
Ultrasound image (bitmap format) showing hypoechoic malignant breast lesion.

This study is a machine learning–based retrospective diagnostic classification. Ultrasound images of 100 malignant and 100 benign breast lesions were downloaded from an open-access repository [7]. The images were in bitmap format, and the size ranged from 7 to 33 kB (Figure 1).

The images were then loaded in ImageJ software (Wayne Rasband). The image despeckling was done to improve the contrast resolution of the images because ultrasonogram images are known to have speckle noise [8]. 

**Figure 2:**
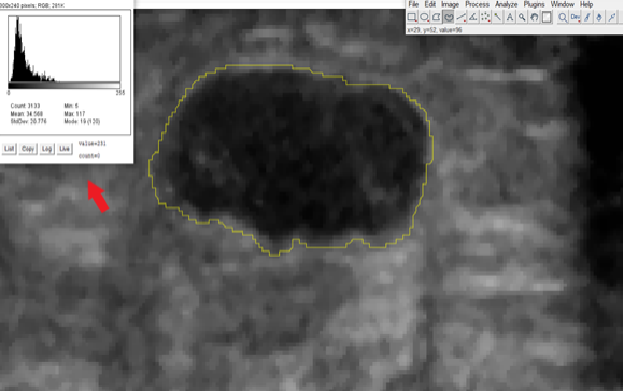
Despeckled ultrasound image of a hypoechoic malignant breast lesion. The image shows the freehand region of interest drawn over the lesion using ImageJ software. Gray scale intensity histogram (red arrow) of the lesion showing the mean, median, and SD values.

The region of interest (ROI) was drawn over the breast lesions in all 200 images by a board-certified radiologist, and the gray scale intensity histogram values were extracted (Figure 2).

The values were entered in a data sheet and were imported to the MATLAB R2020b software (MathWorks).

A total of 200 histograms values were divided by automated randomization available in the software into a training set containing 70% (n=140) of the total images, a validation set containing 15% (n=30) of the total images, and a test set containing 15% (n=30) of the total images.

**Figure 3:**
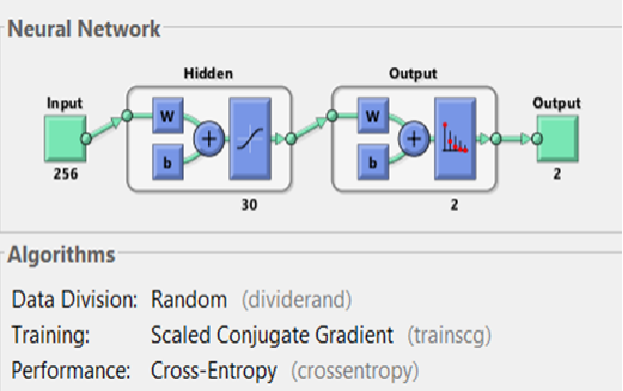
Graphical diagram of the neural network in MATLAB 2020b used for the study with algorithms used for training and performance.

**Figure 4:**
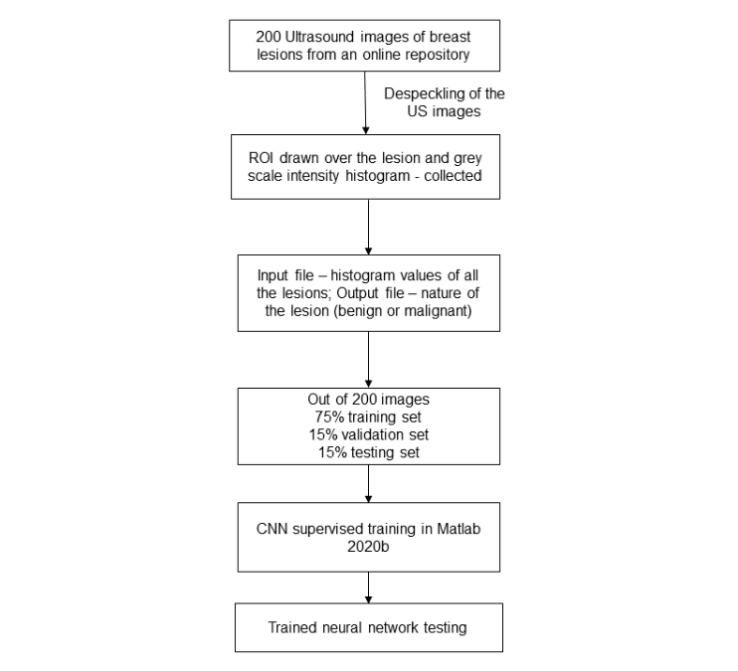
Flowchart describing the workflow of the CNN training and testing performed in the study. CNN: convolutional neural network; ROI: region of interest.

The in-built application of MATLAB R2020b named neural net pattern recognition was used. It is a two-layer feed-forward network with sigmoid hidden and softmax output neurons. The network was trained with scaled conjugate backpropagation available in the software. In our study, we used 30 hidden neurons (Figure 3) [9]. An input file containing the gray scale intensity histogram values (256 values) was fed to the neural network, and a target file containing the output as either malignant or benign was loaded. Supervised training was initiated, and the results were obtained. The flowchart of the methodology is given in Figure 4.

## Results

The supervised training was completed in ~1 second. The training of the CNN took 20 iterations (1 iteration=1 epoch in our study) with 6 validation checks.

**Figure 5:**
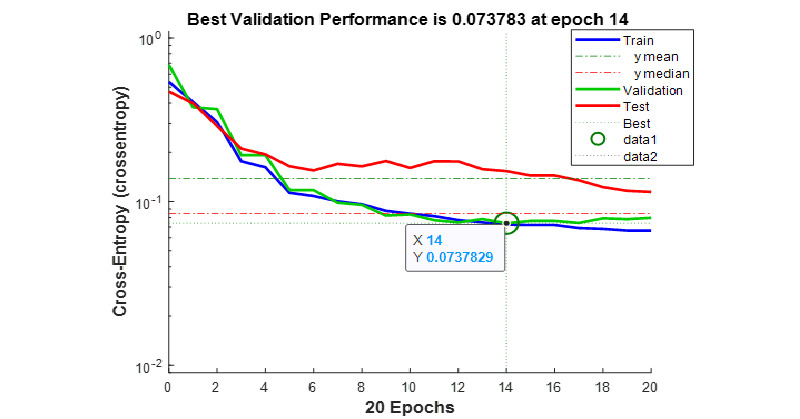
Performance graph of the convolutional neural network training (x-axis: epoch; y-axis: cross-entropy). The best validation performance achieved was 0.0737829 at the 14th epoch. The mean performance was 0.1381, and the median was 0.08445. The red graph shows the performance of the test data set (70% of the data).

The performance of the CNN was measured using cross entropy as a parameter, and the best validation performance was 0.073783, achieved at the 14th epoch (Figure 5).

**Figure 6:**
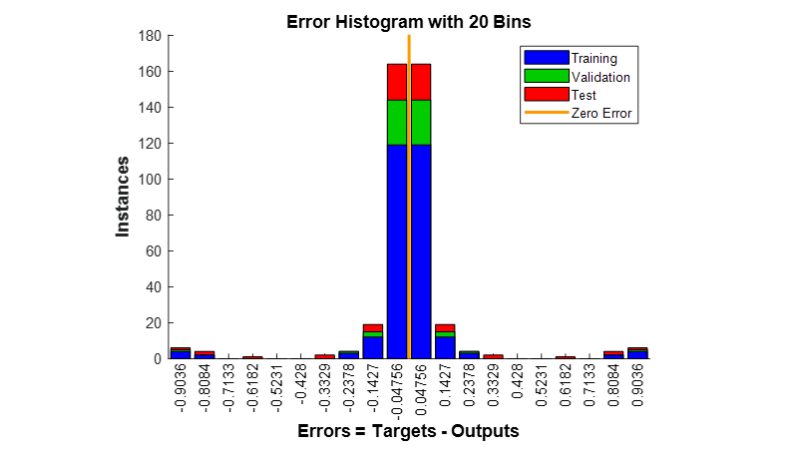
Error histogram with 20 bins (x-axis: error values; y-axis: instances). The histogram shows that zero error lies between the two bins with center error values –0.04756 and 0.04756. The bins with center error values –0.04756 and 0.04756 show the majority of the data fed to the convolutional neural network having error values in that range followed by bins with center error values –0.1427 and 0.1427.

The error histogram exhibiting the number of errors committed by the CNN during the training in each set was acquired (Figure 6).

The results of the training were derived, and the trained neural network was tested using the same data set. The confusion matrix and ROC of the results achieved by the trained neural network was plotted.

**Figure 7:**
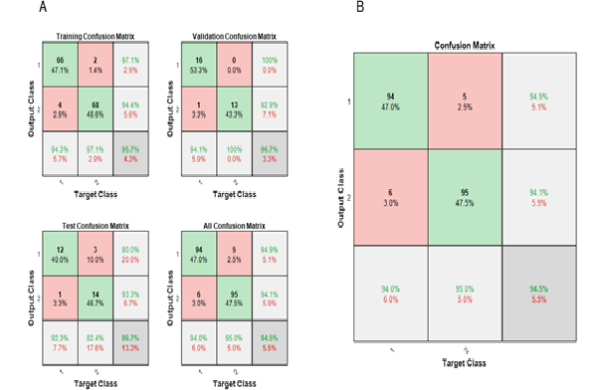
A) Confusion matrices obtained by training the convolutional neural network (CNN) of the training data, validation data, test data, and all the data sets combined. B) Confusion matrix of the trained CNN exhibiting an accuracy of 94.5% and precision of 94%.

**Figure 8:**
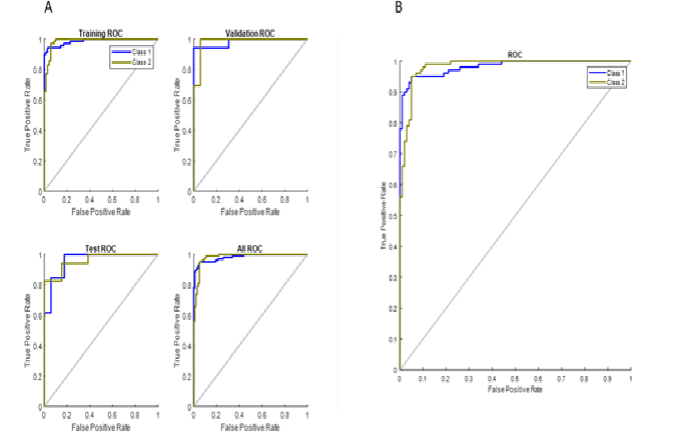
A) ROCs of the training set, validation set, test set, and all sets combined (Class 1: benign; Class 2: malignant). B) ROC of the trained convolutional neural network showing excellent performance with detection rate. ROC: receiver operating curve.

The following describes the values useful for the clinicians in making the diagnostic decision. During training, the CNN on the testing data set showed a sensitivity of 80.0% and a specificity of 93.3%. The accuracy and precision were 86.7% and 92.3%, respectively. The trained neural network, which was tested on the whole data set, showed good results. The sensitivity was 94.9% and the specificity was 94.1%. The negative predictive value and precision of the trained CNN were 95% and 94%, respectively, with an accuracy of 94.5% (Figure 7). The receiver operating curves of the CNN on various data sets during training and the trained CNN, with class 1 as benign breast lesion and class 2 as malignant breast lesion, plotted in the x-axis as the false-positive rate and y-axis as true-positive rate are shown (Figure 8).

## Discussion

Ultrasonograms can be used to define the morphological features of a lesion, and the lesion is reported with details of shape, margin, echo pattern, location, and posterior acoustic characteristics [10]. Terms used for reporting echo findings are subjective and qualitative. In a study conducted by Rahbar et al [11], malignancy was detected in 67% of the lesions with spiculated margins. Of all the lesions, 71% of the hypoechoic lesions and 100% of the hyperechoic lesions turned out to be benign. The US Breast Imaging Reporting and Data System was created to standardize breast US reporting and thereby categorizing breast lesions based on their risk of being a malignancy [12]. Positive predictive values of US features—spiculated margin and irregular shape—were 86% and 62%, respectively, in a study conducted with 403 patients, among which 35% had malignancy. Hyperechoic patterns were not present in any of the malignant lesions in this study [13]. Histogram analysis of gray scale intensity is a quantitative measure of the echo pattern in a lesion, hence can provide objective assessment of the lesion. Erol et al [14] used lesion echogenicity ratios to differentiate between malignant and benign lesions. The mean lesion echogenicity ratio values for benign lesions was 1.63 (SD 0.41) and for malignant lesions was 3.1 (SD 0.87), and the study showed statistically significant difference between malignant and benign lesions.

Machine learning algorithms to diagnose malignant lesions is a highly pursued research topic. A study using a fuzzy support vector machine analyzed eight textural features, three fractal dimensions, and two histogram-based features in identifying a malignant breast lesion in 87 cases reported an accuracy, sensitivity, specificity, precision predictive value, and negative predictive value of 94.25%, 91.67%, 96.08%, 94.29%, and 94.23%, respectively [15]. They analyzed mean, variance, skewness, kurtosis, energy, and entropy of the histogram values using stepwise regression and found out that variance and entropy were the two histogram-based optimal variables that will be useful in diagnosing malignancy. A study by Wang et al [16] used a multi-view CNN and had a sensitivity of 88.6% and specificity of 87.6% in detecting malignancy in ultrasonogram images of 316 breast lesions in two views.

Gray scale intensity values as a sole predictor of malignancy with the help of neural networks was explored in this study. Our study showed an extraordinary performance with an accuracy of 94.5% and precision of 94%, which is slightly higher than in the study by Shi et al [15]. The advantages of our study were that only gray scale histogram values were used to diagnose malignancy, which is easy and convenient to collect, making it easier to reproduce, and that a simple neural network was used with a training duration of ~1 second, making it a viable option in low-resource settings with limited professionals.

The limitations of this study were that US acquisition parameters were not mentioned in the data set, which makes it difficult to standardize the protocol to the general population since US imaging parameters might vary from place to place, and ROIs drawn by different people can vary, which can affect the histogram values, but the effect will be minimal since CNN analyzes the skewness, entropy, variance, kurtosis, and energy of the gray scale intensity values.

## Abbreviations

CNN: convolutional neural network
ROI: region of interest
